# Infrared Spectroscopy for Variety Identification and Authenticity Analysis of Tobacco Samples

**DOI:** 10.3390/s26082544

**Published:** 2026-04-20

**Authors:** Eric Deconinck, Imad Adahchour, Yasmina Naïmi, Maarten Dill

**Affiliations:** Medicines and Health Products, Scientific Direction Physical and Chemical Health Risks, Sciensano, J. Wytsmanstreet 14, B-1050 Brussels, Belgium; imad19982009@hotmail.com (I.A.); yasmina.naimi@sciensano.be (Y.N.); maarten.dill@gmail.com (M.D.)

**Keywords:** authenticity checking, infrared spectroscopy, tobacco products, partial least squares

## Abstract

**Highlights:**

**What are the main findings?**

- Variety identification in tobacco blends is feasible using (N)IR spectroscopy and PLS.
- Proportion estimation of a specific variety using NIR and PLS gave less than 5% errors.

**What are the implications of the main findings?**

- The presented approach proved its utility in the analysis of five commercial samples.
- The approach is valuable for law enforcement in the context of the fight against fraud and counterfeiting.

**Abstract:**

In authenticity checking of tobacco products, the identification of the varieties present is of primary importance. Nowadays the detection of illegal tobacco products is often based on package analysis and administrative verification, sometimes complemented with laboratory analysis. In this study an approach based on IR spectroscopy (MID-IR and NIR) for the identification of tobacco varieties in tobacco blends is proposed. Therefore, different blends were prepared, spectra were measured, and binary PLS-DA models were created. All models were evaluated and compared for their predictive performance, using both cross-validation (internal validation) and an external test set. For the best-performing model for each analyte the limit of detection was estimated. Finally, quantitative models were created to estimate the relative amount of one of the targeted varieties in the mixtures and a proof of concept using five commercial tobacco blends was performed. NIR proved to outperform MID-IR with maximum values of correct classification rate, precision, specificity, and accuracy for four varieties and only one misclassification for the two remaining ones. Indicative limit of detection values were obtained between 1 and 8%. Quantitative errors were all smaller than 5%. These values as well as the application to commercial samples proved the feasibility of the presented approach and its potential value as tool in the fight against fraud and counterfeited tobacco products.

## 1. Introduction

Tobacco products are among the most counterfeited products in the world. They are generally composed of a blend of different tobacco varieties and some additives for enhancing sensory experience [1]. This blend defines the characteristic aroma of a specific commercial blend or product. Since counterfeiters become constantly better in the forgery of packaging and graphics, as well as accompanying documentation, the determination of the variety composition of products could be a valuable tool in the authenticity check of products.

Besides the fight against counterfeiting and fraud, the blend of a commercial product determines the aroma and is therefore an important quality parameter during production in the tobacco industry. Most research on the determination of varieties, blending proportion analysis, and the characteristic profiles was done in this context. Classical techniques used are chromatographic based chemical analysis. Gas chromatography hyphenated with different types of mass spectrometry (MS) is used for the determination of volatile compounds and aromatic profiles [2,3,4,5]. Liquid chromatography combined with MS is more used for metabolomics-oriented analysis [3]. Besides chromatography, physical detection through microscopic observation remains very important [6,7,8]. Although these methods could be used for authenticity determination, they are limited to the use in a laboratory and necessitate a temporary quarantine of products at customs. Moreover, these methods are relatively expensive and labor intensive [8,9].

Spectroscopic techniques are drawing attention in the analysis of tobacco products. Both mid- and near-infrared spectroscopy (MID-IR and NIR) were, for instance, applied for the detection and quantitative estimation of regulated additives in tobacco products [10,11] and for adulteration of products with cannabis [12]. In quality control of tobacco products, IR was already applied for different purposes, i.e., for the determination of moisture content and different chemical compounds, for example, nicotine, sugar, chlorophyll, total nitrogen, and alkaloid content [13,14,15,16,17,18,19], to evaluate the quality and the maturity level of tobacco leaves, to determine/confirm their species or origin [17,18,20,21,22,23,24], and to assure batch-to-batch consistency [8,25]. In all applications, multivariate modelling and calibration techniques were applied in order to interpret the spectra and to create predictive models. Multivariate tools are necessary since the measured spectra of herbal material are complex and difficult to interpret with simple library comparison or visual interpretation.

Industry-related research in quality control shows the potential of IR in the analysis of tobacco products and also in the authenticity determination performed by customs officers or inspection services. Several papers were published on the discrimination of tobacco leaves and products based on their quality grading [23,24,26,27] and the link with their chemical composition [27], the position of the leaves on the plants, and even the position of the plant in the field during cultivation [24]. Based on this, IR could be used to check if the quality of the product corresponds to what it is sold for. More specific, in the context of authenticity, Cao et al. [1] employed NIR spectroscopy for the authentication of five commercial tobacco brands. The focus of the authors was on the classification/discrimination of different brands. However, this is not a situation that is encountered by customs and inspection officers. Indeed, these officers do not dispose of reference samples of all brands with which they could compare the suspected samples. The information that is available is the detailed composition of the tobacco products allowed on the market. In the European Union (EU), for example, manufacturers are obliged to annually notify all tobacco products, providing detailed information on the ingredients through the EU Common Entry Gate (EU-CEG) [28].

This paper focuses on the evaluation of MID-IR and NIR, combined with partial least squares (PLS) modelling for the identification and the proportion estimation of targeted tobacco varieties in mixtures of tobacco varieties. The idea is to present a practical approach for authenticity checking and detection of falsified products to decide whether or not to seize products, which can be easily transferred to portable spectrophotometers. The latter is a key feature for use in the field at customs or inspection sites. Six different pure tobacco varieties were selected, and 85 mixtures were prepared in concentrations ranging from 1 to 100% for each of the selected varieties. Both types of spectra were measured for all prepared mixtures and five commercial tobacco products bought on the Belgian market. The collected spectral data was further subjected to a series of data pre-treatment techniques before PLS modelling. PLS-discriminant analysis (PLS-DA) was used to create binary classification models in order to identify the targeted varieties in the mixtures. PLS regression was applied on the sets of mixtures positive for a specific variety for proportion estimation. Models were validated using an independent test set. Limits of detection were estimated based on the lowest-proportion mixture of each variety classified as positive by the selected models.

The novelty of the approach lies in the focus on the identification and the quantitative estimation of specific tobacco varieties in commercial blends. This allows us to apply different models to all kind of commercial products claiming to contain the targeted varieties. This is in contrast to the majority of the literature, which focuses rather on the analysis of one particular compound or variety in the context of batch-to-batch reproducibility or the authentication of a finished product [8,13,14,25]. Moreover, to our knowledge, binary models, targeting one variety at a time, were never used in this context. This binary approach allows flexibility and easier broadening of the scope, for example, when a supplementary variety has to be added.

## 2. Materials and Methods

### 2.1. Samples and References

The certified references for six pure varieties (flue-cured (RT2), oriental (RT3), burley (RT4), dark air-cured (RT5) (notable for elevated levels of tobacco-specific nitrosamines), dark air-cured (RT9), and dark fire-cured (RT10) tobacco) were purchased from the Kentucky Tobacco Research and Development Center (Lexington, KY, USA). Five commercial samples were purchased at a regular tobacco shop in Leuven, Belgium. The selection of these products was based on the popularity of the products, according to the shop’s owner. For confidentiality reasons, the brands and product names cannot be revealed.

In order to create the data necessary for modelling, 55 mixtures were prepared using the six pure varieties according to standard pharmaceutical practices using a mortar and pestle. The six varieties were mixed in equal quantities to create all possible binary, tertiary, quaternary, and quinary mixtures, resulting in mixtures containing each variety in concentrations of 0, 20, 25, 33.3, and 50%. Finally, a mixture was prepared containing all six considered varieties, resulting in a sample containing 16.7% of each. Considering that the pure varieties were also taken into account, the final sample set of 61 samples (55 mixtures + 6 pure varieties) covered, for each variety, the range of 0 to 100%. Additionally the six quinary mixtures were used to prepare, for each variety, triturations containing 1, 2, 5, and 8% of the variety not included in the quinary mixture used. These mixtures were used for the estimation of the limit of detection of the binary classification models, as well as for regression purposes.

After preparation, all mixtures were stored in airtight containers at −20 °C.

### 2.2. Data Acquisition

#### 2.2.1. Mid-Infrared Spectroscopy (MID-IR)

MID-IR spectra were recorded in the range of 4000 to 400 cm^−1^ using a Nicolet iS1 FTIR spectrometer (ThermoFisher Scientific, Waltham, MA, USA) equipped with a Smart iTR accessory and a deuterated tryglycine sulphate (DTGS) detector. The qualification of the instrument, both at intervals and in use, was performed according to the criteria of the OMCL guideline issued by the European Directorate of Quality of Medicines and Healthcare (EDQM) [29]. This comprised a weekly calibration of the Smart iTR module using a singular-bounce diamond crystal with a polystyrene film standard, cleaning of the crystal between each measurement using a methanol-soaked soft tissue, followed by brief air-drying and a blank measurement between each sample. The latter was performed to check for contamination and carry-over effects. All measured spectra were the result of 32 co-added scans, which were measured at a resolution of 4 cm^−1^. Data collection and simple data pre-treatment, like baseline correction, was performed using the Omnic software, version 9.9.535 (ThermoFisher Scientific, Madison, WI, USA).

All references, mixtures, and samples were measured directly by applying an adequate amount onto the crystal, without further sample preparation, and by applying adequate pressure to ensure adequate layer thickness.

#### 2.2.2. Near-Infrared Spectroscopy (NIR)

NIR spectra were recorded in the range of 10,000 to 4000 cm^−1^ using a Frontier MID-IR/NIR Spectrometer (PerkinElmer, Waltham, MA, USA). The spectra were recorded in reflectance mode, employing the NIR Reflectance Accessory. Each measured spectrum is the result of 16 co-added scans recorded at a resolution of 8 cm^−1^. Between each sample, background spectra were recorded by means of a diffuse reflector from PerkinElmer. The Spectrum 10 Spectroscopy software (PerkinElmer, Waltham, MA, USA) was used for the collection of data, as well as to perform background subtraction and arithmetic corrections to account for background effects. Monthly and weekly checks and calibration were performed, similarly to the MID-IR instrument, following the OMCL guideline in the context of our ISO17025 certification [29].

Different analytes were brought in adequate glass vials up to a height of 1.5 to 2 cm. The measurements were performed without further sample preparation.

### 2.3. Data Analysis

#### 2.3.1. Data Preparation and Pre-Treatment

For MID-IR, only the fingerprint region from 650 to 2000 cm^−1^ was considered for data analysis. The first part of the spectrum (4000–1999 cm^−1^) was cut due to the atypical nature of the spectrum, mainly due to the presence of water in the samples. The region of 649–400 cm^−1^ showed no significant absorbance and only introduced noise into the data. For the recorded NIR spectra, the whole spectrum was taken into account.

Before multivariate modelling, the spectral data should be pre-treated in order to reduce variations due to external factors (environmental, instrument related, etc.), not related to the samples under investigation. Since the development of a modelling method is composed of the selection of the best combination of data pre-treatments and modelling methods, six different pre-treatment approaches were explored. The first approach consisted of autoscaling, correcting scale differences within the data that could influence both the selection and the importance of wavelengths during modelling [30]. The second approach involved the normalization procedure, signal normal variate (SNV), which eliminates variation in the data due to the measurement itself (variations in path length, scattering, or in the detector) [30]. Also, the application of the first and second derivative was explored. These techniques eliminate the background from the spectra and accentuate the spectral framework, highlighting the differences between the spectra and between the samples [30]. The derivatives were calculated with the Savitzky–Golay method using a second order polynomial and a window size of 17 [31]. The last two approaches were the combinations of SNV, followed respectively by the first and second derivative.

All multivariate models should be validated. This can be partially done using cross-validation (CV), which has often served a double purpose in PLS modelling. CV serves both to select the optimal number of latent variables and to estimate the performance of the model, the so called internal validation. However, CV might overestimate the performance of the model; therefore, external validation using an external test set not used in the calculation of the models is necessary [30]. The duplex algorithm was used in this study in order to select a representative test set covering the spectral data space of the sample set. Duplex is based on a pairwise sample selection, iteratively appointing the samples with the highest Euclidean distance between them in the original spectral dataspace to a first set, the samples with the second-highest Euclidean distance to a second set, the third pair to the first set, and so on, until the requested number of samples in the second set is attained. The second set forms the external test set, while the first set, combined with the remaining samples, was used for modelling [32]. In this study, a test set of 20% of the samples was selected for validation, corresponding to 16 samples for classification and 11 samples in regression. Latent variable selection and internal validation was performed using 10-fold cross-validation. The models were evaluated for their performance based on the root mean squared error of cross-validation (RMSECV) and the root mean squared error of prediction (RMSEP) of the external test set. These values are expressed as correct classification rates (CCRs) for the classification models and as percentages for the proportion estimation of the varieties in the samples (regression). For quantitative models, the residual predictive deviation (RPD) and the range error ratio (RER) were calculated. RPD was calculated as the ratio between the standard deviation of the reference values in the test set and the RMSEP. Models with RPD values below 1.5 are considered poor, those with values between 1.5 and 2.0 as allowing some rough screening, those with values between 2.0 and 2.5 as allowing approximate predictions, those with values between 2.5 and 3.0 as good, and those with values higher than 3.0 as excellent. RER was calculated as the ratio between the range of reference values in the test set and the RMSEP. RER values higher than 10 point at excellent models, those with values between 5 and 10 point at good or acceptable models, and those with values below 5 point at poor models [33].

#### 2.3.2. Partial Least Squares (PLS) Analysis

PLS analysis is one of the most applied multivariate modelling techniques across a broad variety of domains. It is a supervised projection technique that defines new latent variables as linear combinations of the manifest variables, such that they represent the highest (PLS factor 1) or highest remaining (PLS factor 2 and further) co-variance with the response. Therefore, the number of dimensions in the data set is reduced, and the highest importance in the model is given to the manifest variables showing the highest co-variance with the response variable [30].

PLS was originally developed for regression purposes, though when combining it with discriminant analysis (PLS-DA), it can also be used for classification modelling [30].

In this study, PLS-DA was applied to create binary models for each of the targeted varieties separately. PLS was used for the quantitative estimation of the proportion of a specific variety in the mixtures, using only the samples positive for the variety under investigation. For both types of models, 10-fold CV was used for the selection of the complexity (number of latent variables) of the model.

#### 2.3.3. Software

All data processing was performed using Matlab R2019b. All algorithms applied were part of the ChemoAC toolbox, version 4.1, developed by the ChemoAC consortium in Brussels, Belgium.

## 3. Results

### 3.1. Recorded Spectra

**Figure 1:**
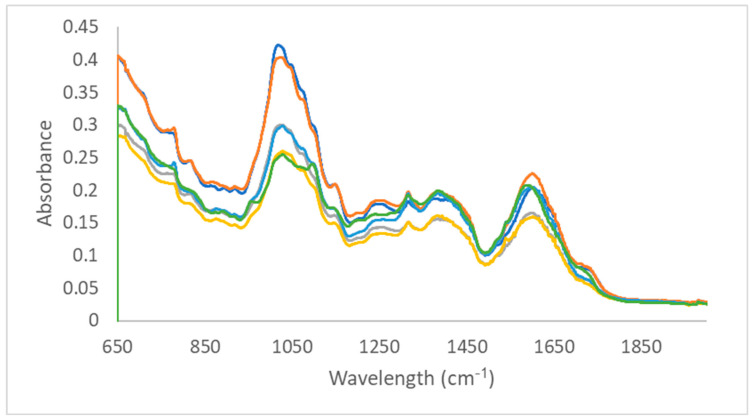
MID-IR spectra measured for the six pure varieties of tobacco (dark blue: flue-cured (RT2); orange: oriental (RT3); grey: burley (RT4); yellow: dark air-cured (RT5); light blue: dark air-cured (RT9); green: dark fire-cured (RT10) tobacco).

Figure 1 shows the fingerprint region of the MID-IR spectra recorded for the six varieties in their pure form.

From Figure 1, it can be observed that a visual differentiation between the varieties is very difficult. This is normal, since all six spectra are measured for varieties of the same plant. In order to catch the small differences, supervised modelling is necessary, especially to identify one of the varieties in mixtures. Although difficult to interpret, some characteristic regions can be identified from the spectra in Figure 1. The region around 1000 cm^−1^ can be associated with out-of-plane C-H bending, typically for aromatic rings present in phenolic compounds. The region between 1100 and 1300 cm^−1^ can be linked to C-O stretching vibrations, typical for alcohols, ethers, and ester, common functional groups in terpenes and flavonoids. The region between 1550 and 1700 cm^−1^ can be either linked to C-C stretching in an aromatic ring or N-H bending in compounds containing a primary amine [34].

**Figure 2:**
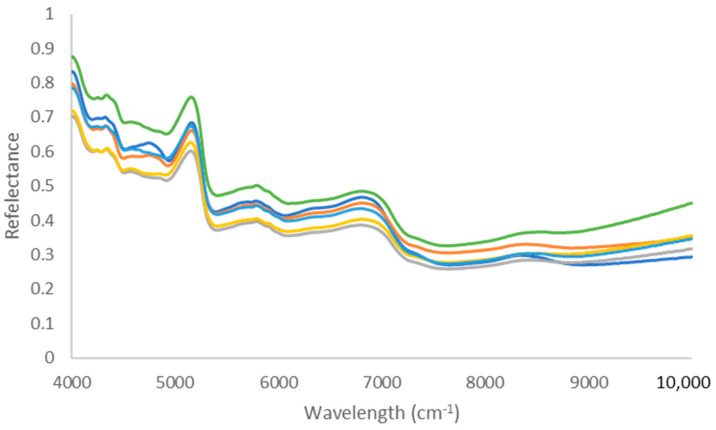
NIR spectra measured for the six pure varieties of tobacco (dark blue: flue-cured (RT2); orange: oriental (RT3); grey: burley (RT4); yellow: dark air-cured (RT5); light blue: dark air-cured (RT9); green: dark fire-cured (RT10) tobacco).

Similarly to MID-IR, Figure 2 shows the NIR spectra recorded for the six varieties.

Also for the NIR spectra, a visual differentiation between the six varieties is nearly impossible, as shown by the spectra presented in Figure 2. However, four regions of the spectra are worth mentioning here. The first is the region between 4250 and 4500 cm^−1^, corresponding to O-H and C-O stretch combination, CH_2_ deformation, C-H bend second overtones, C-H stretch, CH_2_ deformation combination, C-H stretch, CH_2_ deformation combination, and CH_2_ bend second overtones. In this region, these signals can be attributed to the corresponding overtones in cellulose [25]. The second region is the one around 5000 cm^−1^, representing N-H stretch and N-H bend combination, as well as C=O stretch second overtones. The third region (5500–6000 cm^−1^) corresponds to the O-H stretch/C-O stretch second overtone combination, while the fourth region (6500–7000 cm^−1^) corresponds to the O-H and N-H stretch first overtones and possibly also the C=O stretch third overtone [35]. These last signals can be attributed to the presence of nicotine and polyphenolic compounds [9].

### 3.2. Qualitative Models

#### 3.2.1. MID-IR Data

As mentioned before, the recorded spectra were all cut to retain only the fingerprint region. Afterwards, the data was split into test and training set using the duplex algorithm. The ratios for positive vs. negative samples in the test set were, respectively, 9/7 for flue-cured (RT2), 7/9 for oriental (RT3), 7/9 for burley (RT5), 9/7 for dark air-cured (RT5), 6/10 for dark air-cured (RT9), and 9/7 for dark fire-cured (RT10).

The training sets were used to create binary models using PLS-DA for each variety separately. Binary models were chosen since a set of models allows more flexibility in adding new varieties in the future, it requires less computational power, and it often performs better in identifying several targeted analytes in a single sample.

For each variety, six PLS-DA models were built and validated using the external test set using the six different data pre-treatment approaches described above. For each model, the RMSECV values obtained through 10-fold CV were used to select the optimal number of latent variables. In case of equal RMSECV values, the less complex model was chosen as optimal. The selection of the best model (pre-treatment) for each variety was primarily based on the RMSEP for the test set, and only in case of similar RMSEP, the values of CV were taken into account. Table 1 and Table 2 give an overview of the performance parameters of the best-performing model for each of the varieties.

When investigating the misclassifications presented in Table 1, it can be seen that for the flue-cured (RT2) variety, a 100% CCR for the external test set was obtained. Although this is the most important feature, it can be seen that during 10-fold CV, five samples were misclassified, from which two were false negatives and three were false positives. This is also reflected by the precision, specificity, and sensitivity values in Table 2. Both false-negative samples contained a proportion of 33.3% of flue-cured (RT2) and thus, like the other false negatives, these misclassifications are probably due to random modelling errors or slight variations in the spectra that were not filtered by the pre-treatment. It should be noted that poorer performance in CV often occurs due to the fact that, at each iteration, 10% of the training set is taken out of the modelling calculations, and thus the models are based on less information than the final model used for the test set. This is especially relevant for the relatively small sample sets, such as those used in this study.

For oriental 5 (RT3), one false-negative sample and one false-positive sample were observed in the test set, while in cross-validation, four false-negatives and four false positives were recorded. Here, random errors may also be the cause, since in both cross-validation and the test set, the false-negative samples had proportions of oriental (RT3) in the range of 20 to 33.3%.

The burley (RT4) variety seemed the most difficult to model, with five misclassifications in the external test set. Two samples were false positives and three were false negatives. In CV, four false negatives and four false positives were observed.

For the dark air-cured (RT5) variety, all samples of the external test set were correctly classified. During CV, three false negatives and three false positives were observed. The false negatives showed proportions of 33.3 to 50% of the targeted variety.

Three misclassifications were obtained for the dark air-cured (RT9) variety in the test set with one false-negative sample (16.7%) and two false-positives. In CV, however, 14 misclassified samples (seven false-negatives/seven false positives) were observed, showing the difficulty of modelling this variety. Again, the whole range of proportions was covered in the false-negative samples. It was also observed that the false positives were not immediately related to the presence of the dark air-cured (RT5) variety and vice versa. One could think that false positive results could have occurred due to the presence of the other related variety, though this was not the case, since several false positives were negative for both varieties.

Finally, the model for the dark fired-cured (RT10) variety showed one false-negative (33.3%) sample in the external test set and four false negatives and four false positives, respectively, during CV.

After modelling, the score plots obtained with the three first PLS factors were explored. However, no clear clustering could be observed based on these first three latent variables. Also, when exploring the loadings of the different PLS factors included in the respective models, no clear region could be identified as responsible for the classification. The latter was to be expected based on the complexity of the matrix and the fact that this study tries to distinguish varieties of the same plant.

As mentioned before, supplementary mixtures using the quinary blends were used to prepare samples with respectively proportions of the targeted varieties at 1, 2, 5 and 8%. The goal was to define a kind of limit of detection for each selected model, estimating that the lowest proportion classified as positive is the approximative limit of detection. However, due to the complexity of the matrix, these should only be considered as indicative, since the matrices presented here are only considering the six targeted varieties of this study. The estimated limits of detection of the selected models were 1% for the flue-cured (RT2), burley (RT4), and dark air-cured (RT5) varieties, 5% for dark air-cured (RT9) varieties, and 8% for oriental (RT3) and dark fire-cured (RT10) varieties.

#### 3.2.2. NIR Data

The recorded spectra were used as such and submitted to the duplex algorithm for the selection of the external test sets. The ratios for positive vs. negative samples in the test set were 9/7 for the flue-cured (RT2), dark air cured (RT9), and dark fire cured (RT10) varieties and 7/9 for oriental (RT3), burley (RT4) and dark air-cured (RT5) varieties.

Exactly the same modelling and selection approaches as those applied to the MID-IR data were followed, and the performance parameters of each of the selected models are given in Table 1 and Table 2.

Exploring the CCR values in Table 1 shows that, for three varieties (flue-cured (RT2), burley (RT4), and dark air-cured (RT5)), no misclassifications were observed neither in CV nor in the test set. The selected model for the oriental (RT3) variety also showed 100% CCR for the test set; however, three false-negative samples were observed during CV. These samples were mixtures, containing respectively 25, 25, and 16.7% of this variety. The high quality of the selected models is also reflected by the values of precision, specificity, and sensitivity, as given in Table 2.

For the two remaining varieties, very high CCR values were obtained, with one misclassification in the test set and four during CV four each. For the dark air-cured (RT9) variety model, the misclassified sample in the test set was false-negative, containing 50% of the targeted variety, probably due to random error. During CV, two false negatives and two false positives were found. The false negatives contained respectively 33.3 and 16.7% of dark air-cured (RT9) variety. The model for the dark fire-cured (RT10) variety showed a false-positive sample in the test set and four false negatives during cross-validation. From the latter samples, three contained 33.3% of the targeted variety and one contained 25%.

As for MID-IR, the score plots and loading plots of the three first PLS factors for each of the selected models were explored. For the flue-cured (RT2) variety, a tendency for separation could be observed along PLS factor 2 and 3, pointing at a higher importance of the region at the beginning of the spectrum, from 4000 to 4500 cm^−1^, which should correspond to C-H combination bands and overtones (CH3- and CH2-groups), as well as some C-N vibrations. For other models, such trend could not be observed, based on the three first PLS factors. When exploring the loadings, it seems that the first part of the spectrum (4000–6000 cm^−1^) carries more weight. Obviously, this part of the spectrum was not enough to differentiate the different varieties, which can also be concluded from the relatively high number of PLS factors selected. This observation is not surprising, taking into account the complexity of the matrices and the fact that the differentiation of different varieties of the same plant was attempted.

When predicting the prepared mixtures with 1, 2, 5, and 8% of the respective varieties, following indicative limits of detection could be obtained: 1% for flue-cured (RT2) and dark air cured (RT9) varieties, 2% for oriental (RT3) and dark air-cured (RT5) varieties, 5% for dark fire-cured (RT10) varieties, and 8% for burley (RT4) varieties.

### 3.3. Quantitative Models

For quantitative purposes, the dataset, composed of the spectra for the 85 prepared mixtures, was split in six different datasets, containing only the samples positive for the respective varieties. The range of covered proportions is 1 to 100%. PLS regression models were built, exploring the same six pre-treatment approaches as those applied in classification, with the % for the variety under investigation serving as the response variable.

Each data set for regression was composed of 54 samples and was again split into training and test sets using the duplex algorithm. Test sets of 11 samples (±20%) were selected, making sure that they all covered the whole range of proportions in the complete set.

#### 3.3.1. MID-IR Data

Table 3 gives an overview of the modelling and performance parameters determined for the best-performing models, selected based on the RMSEP values for the external test set.

Except for the burley variety, all error values fall under the 10% error, which is a value that can be expected when trying to quantify herbal material in complex mixtures with infrared spectroscopy in a broad range of proportions (1 to 100%). Except for the burley variety, the RMSECV values are of a similar order as the errors obtained for the external test set, pointing at the absence of overfitting. For the burley variety, it can be observed that the RMSECV value is more or less half of the RMSEP value, and therefore it can be supposed that the bad performance of this model occurred due to overfitting. Table 3 also presents the RPD and RER values. The RPD values range between 1.5 and 1.9, indicating models suitable for rough screening and for the first selection of samples for seizure or subsequent laboratory analysis. The RER values ranged from 5.1 to 6.3, pointing at acceptable models. Table 3 also gives the R^2^ values for the correlation between the predicted and real values in the samples for both the external test set and CV. These values should always be interpreted with caution, since they only give an indication about correlation and not about absolute or relative errors in the predictions.

For each of the selected models, the loadings on the different PLS factors were explored. Only slight differences between the loadings on the PLS factors could be observed for all models. The spectra are a result of complex matrices, and therefore the modelling is probably based on slight nuances in the spectra in order to model the proportion of the targeted variety in the mixtures. It is therefore not possible to identify specific regions dominating the quantitative models for each variety. This is a direct consequence of the design of the study, namely, the analysis of a specific variety in a mixture of varieties of the same plant.

#### 3.3.2. NIR Data

The modelling and performance parameters for the selected models for each of the varieties are given in Table 3. The models were again selected based on the errors obtained from the external test set.

In general, it can be said that the modelling results, based on the NIR spectral data, are superior to the quantitative models obtained with MID-IR. For five of the six targeted varieties, errors of less than 5% were obtained for the external test set. This was not the case for the dark air-cured (RT9) variety with a mean error of 6.51. Comparing the RMSE values for test set and CV shows that the values are very similar, and so there is no indication of overfitting. Moreover, R^2^ values, calculated for predicted versus real values, vary between 0.74 and 0.93, which indicates that there is a correlation between the predicted and the real values, pointing at well-performing models. The RPD values ranged between 2.1 and 3.2, showing that, depending on the variety, the model allows predictions ranging from approximate to excellent. The RER values range from 7.0 to 11.7, corresponding to models of good to excellent quality. Also, these values illustrate the superior performance of the models calculated based on NIR spectra compared to those based on MID-IR spectra.

Also, for these models, loadings on the different PLS factors were explored. In general, it can be seen that the two regions, one with a range of 4100–4500 cm^−1^ and the other around 5000 cm^−1^, are presumably more important in the quantification of each of the varieties. Though the differences between the models are small, they point at the fact that there are small nuances in this region, which, when combined with other parts in the spectra, make the models able to estimate the proportion of one variety in a mixture of varieties of the same plant. Loadings are therefore very difficult to interpret in this context.

### 3.4. Application to Commercial Samples

Based on the results discussed above, it is clear that the NIR models are performing best, both in terms of qualitative and quantitative purposes. Therefore, the selected models for each of the targeted varieties were applied to the five commercial samples bought at a regular tobacco shop.

Table 4 shows the results of the application of the models to these samples. Quantitative models were only applied to the samples when the qualitative model gave a positive result for the variety under investigation.

The results for the five samples for flue-cured (RT2) tobacco show that all samples were predicted as negative. This corresponds to the data found on the official application for information on smoking products of the Belgian Federal Public Service for Health, Food Chain Security en Environment (FPS) (https://apps.health.belgium.be/smokinginfo/home (last accessed 16 April 2026)). For the oriental (RT3) variety, only samples 1 and 5 were predicted positive, and the quantitative models returned values with absolute differences from the real values of 5.7 and 0.8% (Table 4). According to the FPS, sample 2 should have also contained this variety, but in a relatively low amount, 1.8%, which was at the limit of detection of 2%, which was estimated previously. This is probably the reason why this sample was classified as negative. Sample 3 and 4 were predicted as negative, which corresponds to reality. All samples were predicted positive for the burley (RT4) variety, with absolute differences between real and predicted proportions between 2.7 and 6.1%. Sample 3 was predicted with a negative value, probably due to the very low amount (0.3%) present in this sample. The lowest proportion taken into account for the creation of the model was 1%. In this case, the output from the model was <1% which was also in compliance with the database.

For other three varieties, the results were more difficult to interpret, since the FPS data only mention “dark” and not the type of the dark variety. According to the official data, sample 3 and sample 5 contain a “dark” variety. Based on the models, all samples were negative for dark fire-cured (RT10) varieties, sample 3 was positive for both dark air-cured types, and sample 5 was positive only for the dark air-cured (RT5) variety. Since the quantitative results for samples 3 and 5 for the models of dark air-cured (RT5) variety are near the values found in the FPS database, it could be considered that the samples contained this variety. Sample 3 also tested positive for dark air-cured (RT9) variety with an estimated proportion of 5.2%, which is significantly higher than the 0.4% claimed. Therefore, this could be a prediction error due to the more complex matrix of the commercial product or the high similarity between the two varieties. However, the validation results for the qualitative models of both dark air-cured varieties were excellent, pointing at a clear differentiation between both varieties. Since no data was available on the variety present in sample 3, no further explanation of these results could be found. It is possible that the sample contains RT5, a mixture of RT5 and RT9, or perhaps another dark air-cured variety not taken into account in this proof of concept.

## 4. Discussion

The goal of the presented study was to evaluate the use of infrared spectroscopy for the detection and quantitative estimation of targeted tobacco varieties in commercial tobacco blends in the context of authenticity checking and the fight against counterfeit products. For this purpose, six different tobacco varieties were selected, and mixtures of them were prepared to create and validate PLS models based on both MID-IR spectra and NIR spectra. Binary models were built for each selected variety separately for three reasons: (1) a set of different binary models is easier to update in case it is necessary to add a supplementary variety; a global model predicting different varieties, for example, PLS2 models, would necessitate the complete update of the model; the latter should preferably include the repetition of the recordings of the spectra for all varieties; and binary models are more flexible to work with; (2) binary models are generally performing better in terms of predicting analytes in complex matrices, and they also allow for adding more varieties to the set and better predicting the presence of several analytes in one sample; and (3) binary models are easier to compute and demand less computational time and power, making them more suitable to be implemented with or transferred to portable spectrophotometers.

In order to better compare the performance of the two spectroscopic techniques, exactly the same modelling approaches were followed for both.

PLS was chosen in this study for several reason. First of all, PLS is one of the most applied modelling techniques, and it is often available in the software packages offered with IR spectroscopic instruments, both benchtop and portable. If the multivariate calibration approach is part of the software package of the instrument, it facilitates its implementation and use by customs and law enforcement officers, who are less familiar with chemistry and analysis. Moreover, some papers already explored the use of more recent and complex modelling approaches. Cao et al. [1], for example, explored the use of support vector machines (SVMs), k-Nearest Neighbours (kNN), multilayer perceptron, (MLP), Convolutional Neural Networks (CNNs), and Residual Neural Networks for the classification/identification of five commercial tobacco products. Although some gains in performance could be obtained by using these methods, the results of these techniques were similar to the ones of PLS-DA. It could be debated if the small improvement is worth the higher complexity of the analysis. Xiao et al. [25] did similar work regarding the proportion estimation of four different tobacco components and explored, besides PLS, also SVMs, Gaussian Process Regression (GPR), Bayesian Ridge Regression (BRR), and CNNs. There, the results of the more complex techniques were similar or sometimes even worse than the PLS results. Both authors also explored the use of feature selection methods, and, although in both types of models, some gains could be obtained, the differences in results were small and in some cases even negligeable [1,25]. To conclude, in this study, we decided to work solely with PLS modelling, using the whole spectrum for NIR and the whole fingerprint region for MID-IR.

Following this approach, models were created with the spectral data obtained from both techniques. From Table 1 and Table 3, it could be observed that both techniques performed well in qualitative analysis, though NIR clearly outperformed MID-IR. This trend was also observed for the quantitative models, where MID-IR showed prediction errors under 10%, while for NIR, prediction errors were under 5% for the external test set. These results could explain why almost no papers were encountered using MID-IR for the analysis of the herbal components of tobacco blends. It seems that MID-IR introduces more noise in the model and has difficulty to show small spectral differences between the targeted varieties. Other reasons are more technical, such as the higher amount of product in contact with the IR beam in NIR than in MID-IR and the fact that NIR is penetrating deeper into the analyzed material. These technical aspects make MID-IR more susceptible to inhomogeneities in the samples.

In the context of this study, aimed at the use of IR spectroscopy for authenticity checking and the fight against counterfeit products, the errors obtained are acceptable. As mentioned before, both spectroscopic techniques gave PLS models with low levels of misclassification in the qualitative models and errors in proportion estimation of less than 10% for MID-IR and less than 5% for NIR. These values show that both approaches are suitable for the first triage of the samples at, for example, customs. Indeed, especially when portable instruments are used, the customs officers could use this approach to select the samples to be seized and to be further checked in a laboratory, as well as the ones that raise less suspicion. Here, the proposed methods would signify a gain in efficiency and resource utilization, especially since less laboratory analyses would be required. A supplementary advantage of the spectroscopic techniques is their non-destructive character, allowing the same sample to be used in further laboratory analyses.

If the misclassification ratios and prediction errors on the proportions obtained in this study were compared to some recent examples, where NIR was used for the analysis of herbal components of commercial tobacco blends, the errors could seem high. This is, however, a direct result of the design of the study. For example, Cao et al. [1] found CCR values of 100%, but here, five commercial products were distinguished, which is different from the search for varieties within the mixtures. It should also be kept in mind that commercial products do not only consist of herbal components, but they may also contain some additives, which could result in higher distinctive characteristics detected through spectroscopy. Xiao et al. [25] had quantitative prediction errors less than 1.5%, but the study was focused on the analysis of one specific product. A similar comment can be given for the study performed by Jiang et al. [9]. They also focused on the detection of four tobacco components in one specific brand. Moreover, validation was only performed using leave-one-out CV. The models presented in the present paper are more generally applicable and target each variety individually, without taking the product into account. Moreover, all models were validated using an external test set, and feasibility was demonstrated by applying the best-performing NIR models for each variety to five commercial samples, yielding promising results.

Although promising results were obtained, the study has some limitations. One important weak point is the use of only one batch of pure varieties targeted in this paper. Indeed, tobacco is a natural product, and, therefore, it is susceptible to variations due to climate differences, harvest time, differences in soil, etc. These factors have influence on the concentrations of certain components, as well as on the residual humidity in the product. Those differences will clearly be reflected in the spectra, and they have an influence on the performance of the models. The use of several batches of pure varieties would permit ti include a part of the natural variation. Another point is the use of equally proportion mixtures for modelling, simplifying the matrices of commercial products. Indeed, going through the database of the Belgian FPS, some varieties may be present in amounts as low as 0.5% and others in amounts of 40% in one product. Moreover, additives or flavours are often added, which could interfere with the spectra. They were not present in the set of samples prepared for the calibration of the model.

In short, further optimization of the approach would be necessary in order to increase robustness during practical application at customs or by law enforcement. This optimization step comprises the creation of new models based on sample sets, including mixtures based on multiple batches of the varieties, preferably from different suppliers from different parts of the world. Moreover, a closer match between the modelled mixtures and the realistic amounts found in commercial blends, and taking additives into account could further improve the models and enhance generalizability.

## 5. Conclusions

MID-IR and NIR were explored for the detection and proportion estimation of targeted tobacco varieties in tobacco blends. NIR outperformed MID-IR both in qualitative as quantitative modelling, though, in cases where NIR equipment was not available, the MID-IR models were sufficiently performant for the first triage of samples.

The validation results, as well as the application of the models to five commercial samples, proved the feasibility of the presented approach, as well as its value in the fight against fraud and counterfeiting.

Although the techniques applied are not new, the novelty of the presented approach lies in the use of flexible binary modelling with PLS to model or target one specific tobacco variety in commercial products. The set of binary models and targeted quantitative models can give a first insight in the composition of a product, allowing its application to a wide range of different products. Moreover, the set can be complemented with new binary models in case the scope of the approach needs to be broadened with other varieties or components, for example, additives. In this case no complete validation would be necessary as would be the case for global multi-target models.

In practice, if the shortcomings of this research, like the incorporation of batch-to-batch variability, realistic proportioning, and the evaluation of the influence of additives, are tackled, the presented approach can be transferred to portable devices. This statement is based on the features preselected for conducting the modelling in this paper. Resolutions and data points were chosen as a compromise between spectrum quality and size of the generated data. PLS, often present in software packages sold with instruments, was used and the focus was on binary models, requiring less computational power. The latter is important since, in the field, often no heavy computational computers are available.

This study is a proof of concept working towards a practical application for in-field work by customs and law enforcement workers. This approach would allow them to quickly check if the composition of the product could correspond to what is claimed, as a complement to packaging and administrative controls. This will allow for better triage of samples, providing a more solid basis for the selection of samples for seizure or for subsequent laboratory analysis and resulting, at the end, in resource savings, especially for laboratory-based analysis and in a reduced number of unnecessary blocking of products at customs.

## Figures and Tables

**Table 1:** Overview of the performance values for the selected classification models for each variety, together with complexity and the selected data pre-treatment.

Spectroscopic Technique	Variety	No PLS Factors	Data Pre-Treatment	CCR% (External Test Set)	CCR% (Cross-Validation)
MID-IR	Flue-cured (RT2)	5	2nd derivative	100 (16/16)	88.89 (40/45)
	Oriental (RT3)	5	2nd derivative	87.5 (14/16)	82.22 (37/45)
	Burley (RT4)	7	SNV	68.75 (11/16)	84.44 (38/45)
	Dark air-cured (RT5)	5	autoscaling	100 (16/16)	86.67 (39/45)
	Dark air-cured (RT9)	8	2nd derivative	81.25 (13/16)	68.89 (31/45)
	Dark fire-cured (RT10)	5	autoscaling	93.75 (15/16)	82.22 (37/45)
NIR	Flue-cured (RT2)	15	autoscaling	100 (16/16)	100 (45/45)
	Oriental (RT3)	12	autoscaling	100 (16/16)	93.33 (42/45)
	Burley (RT4)	11	SNV	100 (16/16)	100 (45/45)
	Dark air-cured (RT5)	10	autoscaling	100 (16/16)	100 (45/45)
	Dark air-cured (RT9)	10	SNV	93.75 (15/16)	91.11 (41/45)
	Dark fire-cured (RT10)	4	autoscaling	93.75 (15/16)	91.11 (41/45)

**Table 2:** Precision, specificity, and sensitivity values for the selected models.

Spectroscopic Technique	Variety	Precision (External Test Set/Cross-Validation)	Specificity (External Test Set/Cross-Validation)	Sensitivity (External Test Set/Cross-Validation)
MID-IR	Flue-cured (RT2)	1.0/0.87	1.0/0.87	1.0/0.91
	Oriental (RT3)	0.86/0.83	0.89/0.82	0.86/0.83
	Burley (RT4)	0.67/0.82	0.78/0.75	0.57/0.72
	Dark air-cured (RT5)	1.0/0.86	1.0/0.88	1.0/0.86
	Dark air-cured (RT9)	0.71/0.71	0.80/0.67	0.83/0.67
	Dark fire-cured (RT 10)	1.0/0.80	1.0/0.84	0.89/0.80
NIR	Flue-cured (RT2)	1.0/1.0	1.0/1.0	1.0/1.0
	Oriental (RT3)	1.0/1.0	1.0/1.0	1.0/0.89
	Burley (RT4)	1.0/1.0	1.0/1.0	1.0/1.0
	Dark air-cured (RT5)	1.0/1.0	1.0/1.0	1.0/1.0
	Dark air-cured (RT9)	1.0/0.90	1.0/0.92	0.90/0.90
	Dark fire-cured (RT 10)	0.89/1.0	0.88/1.0	1.0/0.81

**Table 3:** Overview of the performance values for the selected regression models for each variety, together with complexity and the selected data pre-treatment.

Spectroscopic Technique	Variety	No PLS Factors	Data Pre-Treatment	RMSEP (%)	R^2^p	RPD	RER	RMSECV (%)	R^2^cv
MID-IR	Flue-cured (RT2)	4	SNV + 2nd derivative	8.24	0.74	1.7	6.0	8.8	0.70
	Oriental (RT3)	9	autoscaling	9.62	0.62	1.5	5.1	9.93	0.64
	Burley (RT4)	8	SNV + 2nd derivative	12.8	0.77	1.9	6.3	5.91	0.64
	Dark air-cured (RT5)	7	SNV + 1st derivative	9.18	0.81	1.7	5.4	9.86	0.64
	Dark air-cured (RT9)	3	SNV	8.60	0.53	1.5	5.1	10.84	0.66
	Dark fire-cured (RT10)	4	SNV	9.50	0.79	1.5	5.2	8.70	0.71
NIR	Flue-cured (RT2)	11	SNV	3.59	0.92	3.1	11.7	4.13	0.93
	Oriental (RT3)	10	1st derivative	3.40	0.90	2.7	9.0	5.80	0.88
	Burley (RT4)	11	SNV	4.46	0.74	2.1	7.0	8.09	0.76
	Dark air-cured (RT5)	9	SNV	4.83	0.90	2.8	11.7	4.58	0.91
	Dark air-cured (RT9)	4	SNV	6.51	0.78	2.2	7.4	7.33	0.81
	Dark fire-cured (RT10)	5	1st derivative	4.42	0.93	3.2	11.1	6.11	0.85

**Table 4:** Prediction of real samples with the NIR models (“-” means negative in the qualitative models; values between brackets are the values found on https://apps.health.belgium.be/smokinginfo/home (last accessed 16 April 2026)).

Variety	Sample 1	Sample 2	Sample 3	Sample 4	Sample 5
Flue-cured (RT2)	-	-	-	-	-
Oriental (RT3)	18.4% (12.7%)	−(1.8%)	-	-	5.0% (4.2%)
Burley (RT4)	28.3% (22.2%)	36.6% (33.9%)	−7.0% (0.3%)	37.0% (32.2%)	4.1% (8.1%)
Dark air-cured (RT5)	-	-	0.73% (0.4%)	-	12.4% (14.5%)
Dark air-cured (RT9)	-	-	5.2%	-	-
Dark fire-cured (RT 10)	-	-	-	-	-

## Data Availability

The raw data supporting the conclusions of this article will be made available by the authors upon request.

## Abbreviations

The following abbreviations are used in this manuscript:

BRR	Bayesian Ridge Regression
CEG	Common Entry Gate
CNN	Convolutional Neural Networks
CCR	correct classification rate
CV	cross-validation
DA	discriminant analysis
EDQM	European Directorate for Quality of Medicines and Health Care
EU	European Union
FPS	Federal Public Service Health, Security of the Food Chain and Environment
GPR	Gaussian Process Regression
kNN	k-Nearest Neighbours
MID-IR	mid-infrared
MLP	multilayer perceptron
MS	mass spectrometry
NIR	near-infrared
PLS	partial least squares
RMSEC	root mean squared error of calibration
RMSECV	root mean squared error of cross-validation
RMSEP	root mean squared error of prediction
SNV	Signal Normal Variate
SVM	support vector machine
